# Human Leukocyte Antigen Alleles Compatibility and Immunophenotypic Profile Associations in Infertile Couples

**DOI:** 10.7759/cureus.36584

**Published:** 2023-03-23

**Authors:** Georgia Oikonomou, Nikolaos Vlachadis, Vassilios Tsamadias, Irene Lambrinoudaki, Efthymios Deligeoroglou, Nikolaos F Vlahos, Emmanuel Economou

**Affiliations:** 1 Second Department of Obstetrics and Gynecology, National and Kapodistrian University of Athens Medical School, Aretaieio University Hospital, Athens, GRC; 2 Department of Obstetrics and Gynecology, General Hospital of Messinia, Kalamata, GRC; 3 Clinical Laboratory for Therapeutic Individualization, National and Kapodistrian University of Athens Medical School, Aretaieio University Hospital, Athens, GRC; 4 Menopause Unit, National and Kapodistrian University of Athens Medical School, Aretaieio University Hospital, Athens, GRC

**Keywords:** recurrent implantation failure, immunogenetics, ivf-et failure, immuno-clinical profiles, recurrent miscarriage, human leukocyte antigen (hla)

## Abstract

Introduction: The maternal immune system has a major role in the successful embryo implantation and maintenance of the pregnancy. This study aimed to investigate the maternal immunophenotyping profile (percentage of Natural Killer [NK] cells and the CD4/CD8 [cluster designation] ratio in peripheral blood lymphocytes) and the HLA (Human Leukocyte Antigen)-DQA1 alleles sharing in infertile couples.

Methods: This cross-sectional study included 78 women who had experienced at least two spontaneous miscarriages and 110 women with a history of recurrent implantation failures after in vitro fertilization (IVF) or intracytoplasmic sperm injection (ICSI) and embryo transfer (ET) (IVF-ET failures). The NK cell percentage and the CD4/CD8 ratio were determined by flow cytometry. Genotyping of the HLA-DQA1 alleles was carried out for all women and their partners, and couple HLA-DQA1 compatibility was expressed as the percentage of common HLA-DQA1 alleles (totaling 35 alleles) shared between spouses to the sum of the unique alleles observed.

Results: In women with recurrent miscarriages, high values (%) of the NK population with a median (interquartile range [IQR]) of 10.3% (7.7% to 12.5%) and CD4/CD8 ratio (1.7) (1.5 to 2.1) were found. In women with IVF-ET failures, the (%) NK population (10.5%) (8.6% to 12.5%) and CD4/CD8 ratio (1.8) (1.5 to 2.1) were similarly increased (p=0.390, and p=0.490, respectively). The proportion of women with >10% NK cells was 53.8% and 58.2% in women with miscarriages and IVF-ET failures, respectively (p=0.554). The prevalence of HLA-DQA1*5 allele carriage was elevated in women with miscarriages as well as those with IVF-ET failures (52.6% and 61.8%, respectively; p=0.206). The proportion of couples with high (>50%) HLA-DQA1 sharing was 65.4% in the group with miscarriages and 73.6% in the group with IVF-ET failures, respectively (p=0.222). The CD4/CD8 ratio was statistically significantly positively correlated with the (%) NK population in women with IVF-ET failures (rho = 0.297, p=0.002) and with the (%) HLA-DQA1 sharing in the group with miscarriages (rho = 0.266, p=0.019). The couples in which both spouses were carriers of the HLA-DQA1*5 allele had an increased probability of high (>50%) HLA-DQA1 compatibility compared with the couples in which neither of the spouses carried the allele in the miscarriage group (OR = 24.3, 95% CI: 3.0 to 198.9, p<0.001), and the IVF-ET failure group (OR = 10.5, 95% CI: 2.2 to 49.8, p<0.001).

Conclusion: The peripheral NK (%) population and CD4/CD8 ratio, as well as the prevalence of the HLA-DQA1*5 allele, were elevated in women with recurrent miscarriages and IVF-ET failures. Furthermore, these couples with negative reproductive outcomes had a high percentage of HLA-DQA1 allele similarity. The presence of the HLA-DQA1*5 allele in spouses was strongly associated with overall couple HLA-DQA1 compatibility, implying that it could be used as a surrogate marker for assessing overall immunological compatibility in infertile couples.

## Introduction

Miscarriage is the most common complication of pregnancy, occurring in 15%-25% of pregnancies. However, less than 5% of all couples will be confronted with recurrent miscarriages, which is a distinct disorder defined as two or more consecutive early fetal losses prior to the 20th week of gestation [1]. Recurrent miscarriages are a syndrome with a highly heterogeneous etiology. Possible etiological factors include uterine anatomical factors, genetic, infectious, hormonal, and immunological factors, as well as disorders of acquired and inherited thrombophilia. However, the causes of about half of all spontaneous miscarriages remain elusive [1-3].

Recurrent implantation failure is a serious complication of embryo transfer (ET) after treatment of infertility with in vitro fertilization (IVF) or intracytoplasmic sperm injection (ICSI) (IVF-ET failure). The exact definition of recurrent IVF-ET failures remains unclear, but it is generally considered a syndrome in which failures to achieve a clinical pregnancy occurs after at least two good-quality embryo transfers. There is considerable evidence that the above two syndromes share largely common pathogenetic mechanisms [4,5].

Human leukocyte antigen (HLA) genes play a major role in enabling the immune system to recognize “self” versus “non-self” antigens and are divided into classes Ia (HLA-A, -B, -C), Ib (HLA-E, -F, -G, -H), and II (HLA-DM, -DP, -DQ, -DR), which are involved in antigen presentation to CD8 (cluster designation), Natural Killer (NK), and CD4 cells, respectively [6].

Pregnancy is a situation of physiological immunosuppression. As the fetus is a semi-allograft, which escapes maternal immune rejection in normal pregnancy, many studies have investigated whether the HLA system may play a role in unexplained spontaneous abortions [7,8]. Trophoblast antigenic composition is mainly represented by antigens histocompatibility in the II class, including HLA-DQA1 genes, allowing them to be used as immunological markers of recurrent miscarriage incidence. There is evidence that the HLA-DQA1 complex suppresses the maternal immune response required for implantation. It is assumed that in the presence of a homogeneous HLA-DQA1 histocompatibility complex (common for the mother and father), the frequency of recurrent miscarriages increases [9]. Natural Killer (NK) cells are a type of cytotoxic lymphocytes that are thought to be involved in embryo implantation. It is postulated that an increase in NK cells may affect the reproductive outcome, and the proportion of NK cells in the total number of lymphocytes in the blood is used as a potential diagnostic test in women suffering from infertility [10]. CD4 and CD8 T cells are important for the immune response and human reproduction, and the CD4/CD8 ratio is often used to express the immune status of a subject. Evidence suggests that CD4 and CD8 proportions are altered in women with infertility [11].

Considering the potential importance of the above-mentioned factors in human fertility, we aimed at investigating the associations of these immunogenetic and immunophenotypic parameters (NK cell population, CD4/CD8 ratio, as well as HLA-DQA1 genotyping) in two groups of Greek subfertile couples, one with recurrent miscarriages and one with recurrent IVF-ET failures.

## Materials and methods

This cross-sectional study evaluated infertile couples' records who were reviewed in the Clinical Laboratory for Therapeutic Individualization of Aretaieio University Hospital, Athens, Greece. The study was in compliance with the Declaration of Helsinki on medical protocol and ethics. The Aretaieio University Hospital Ethics Committee approved the study protocol (approval number: B-107/30.4.2015), and written informed consent was obtained from all the participants. The study group comprised 78 nulliparous women who had experienced at least two spontaneous miscarriages before the completion of 20 weeks of gestation and 110 nulligravida women who had experienced at least two IVF/ICSI-ET failures with the same partner. All subjects were of Greek origin, healthy, and did not receive any medications during the study period. A thorough medical and obstetric history was obtained for each patient. All cases were referred to our department after primary evaluation according to a standard protocol set up to detect known or putative causes of pregnancy loss, consisting of karyotypes in both parents, male partner spermiogram, ultrasonography examination, hysterosalpingogram and/or hysteroscopy, infection tests, and comprehensive determination of hormonal status.

Requirements for couples to enter the study included extensive laboratory investigation for the exclusion of non-immune etiologies of the miscarriages and for the elimination of other possibly coexisting pathologic factors: anatomical/uterine abnormalities, abnormal karyotype, if available, chronic systemic or reproductive tract infections, abnormal maternal hormonal functions, metabolic diseases, and thrombophilic predisposition. Histology/immunohistochemistry of the placental material of previous spontaneous abortion material was recommended, as well as preimplantation genetic diagnosis, if available, during the IVF/ICSI cycles.

Genotypes for all women and their partners were determined by molecular genetic methods. These techniques are very precise, and the pitfalls of serological methods (cross-reactions, etc.) can be avoided. DNA was extracted from the subjects' leukocytes by a standard phenol/chloroform/proteinase K method. This procedure was followed by polymerase chain reaction (PCR) amplification of 256 bp (exon 1) and 1065 bp (exons 2+3) of the HLA-DQA1 gene. Genotyping of HLA-DQA1 alleles was carried out using the INNO-LiPA DQB PCR-reverse hybridization kit, allowing the discrimination of 35 alleles (Innogenetics, Ghent, Belgium). Couple HLA compatibility was expressed as the percentage of common sharing alleles between couples to the sum of the unique alleles observed (Table 1).

**Table 1:** The 35 alleles used to determine the couple's HLA-DQA1 compatibility.

	Allele
1	DQA1*01:01:01
2	DQA1*01:01:02
3	DQA1*01:02:01
4	DQA1*01:02:02
5	DQA1*01:02:03
6	DQA1*01:02:04
7	DQA1*01:03
8	DQA1*01:04:01
9	DQA1*01:04:02
10	DQA1*01:05
11	DQA1*01:06
12	DQA1*01:07
13	DQA1*02:01
14	DQA1*03:01:01
15	DQA1*03:02
16	DQA1*03:03
17	DQA1*04:01:01
18	DQA1*04:01:02
19	DQA1*04:02
20	DQA1*04:03N
21	DQA1*04:04
22	DQA1*05:01:01
23	DQA1*05:01:02
24	DQA1*05:02
25	DQA1*05:03
26	DQA1*05:04
27	DQA1*05:05
28	DQA1*05:06
29	DQA1*05:07
30	DQA1*05:08
31	DQA1*05:09
32	DQA1*05:10
33	DQA1*06:01:01
34	DQA1*06:01:02
35	DQA1*06:02

Blood samples from all women were obtained at the beginning of the menstrual cycle between menstrual cycle days two and four and were collected in heparinized tubes in the morning under fasting conditions and analyzed within 8h of collection. Aliquots of 100 mcl of blood were placed in flow cytometric tubes (Becton Dickinson, Oxford, UK) and incubated for 15 min at room temperature with mouse anti-human CD16-FITC and anti-human CD56-PE (BD PharMingen, San Diego, CA, USA). Aliquots of 1 ml of Quicklysis lysing solution (Quest Biomedical, UK) were added to each tube and incubated for a further 5 min at room temperature. During the lysis, there were no wash procedures. After the supernatant was removed, 300 mcl of phosphate buffer solution was added to the cells, which were then analyzed by a flow cytometry machine (Beckman Coulter, USA).

Quantitative variables were expressed as mean ± standard deviation (SD) and/or median ± interquartile range (IQR, 25th to 75th percentile), and categorical variables were presented as absolute frequencies and percentages (%). Three parameters (NK cell population, CD4/CD8 ratio, and (%) HLA-DQA1* sharing between partners) were compared between the two groups of subfertile couples (miscarriages vs. IVF-ET failures), as well as according to the carriership of the HLA-DQA1*5 allele. Pearson’s chi-square test was used for frequency comparison. For the quantitative variables, distribution normality was checked using the Kolmogorov-Smirnov and the Shapiro-Wilk tests, and comparisons were performed using the non-parametric Mann-Whitney U test due to significant differences from the normal distribution. The associations were expressed as odds ratios (OR) with a corresponding 95% confidence interval (95% CI), and a p-value <0.05 was considered statistically significant. Statistical analysis was performed using the IBM Corp. Released 2013. IBM SPSS Statistics for Windows, Version 22.0. Armonk, NY: IBM Corp.

## Results

Among a total of 188 infertile women included, there were 78 women with miscarriages, aged 28-40 years (mean: 2.5 ± 0.8 miscarriages, range: 2 to 5, median: 2), and 110 women with IVF-ET failures, aged 28-40 years (mean: 3.9 ± 2.2 IVF-ET failures, range: 2 to 9, median: 3).

In all infertile women, the mean NK cell population was 10.7% (± 3.7%), with a median of 10.4% (8.4% to 12.5%). Comparing the group of women with miscarriages with that of women with IVF-ET failures, the mean percentage of NK cells was 10.4% (± 3.5%) and 11.0% (± 3.8%), respectively, with a median value of 10.3% (7.7% to 12.5%) and 10.5% (8.6% to 12.5%), respectively, without a statistically significant difference (p=0.390) (Table 2).

**Table 2:** The distribution of the (%) NK cell population in women with recurrent miscarriages and IVF-ET failures.

		Total (%)	Miscarriages (%)	IVF-ET Failures (%)
		N=188	N=78	N=110
Mean		10.7	10.4	11.0
Median		10.4	10.3	10.5
SD		3.7	3.5	3.8
Minimum		2.4	2.4	3.2
Maximum		24.3	23.7	24.3
Percentiles	25	8.4	7.7	8.6
	75	12.5	12.5	12.5
Normality test (p-value)	Kolmogorov-Smirnov	<0.001	0.042	0.001
	Shapiro-Wilk	<0.001	0.013	0.001

The proportion of women with >10% NK cells was 106/188 = 56.4% overall, 42/78 = 53.8% in women with miscarriages, and 64/110 = 58.2% in women with IVF-ET failures, with no statistically significant difference (p=0.554).

Overall, the mean value of the CD4/CD8 ratio was 1.8 (± 0.5), with a median value of 1.7 (1.5 to 2.1). Comparing the group of women with miscarriages with the group of women with IVF-ET failures, the mean CD4/CD8 ratios were 1.8 (± 0.5) and 1.8 (± 0.6), respectively, with a median value of 1.7 (1.5 to 2.1) and 1.8 (1.5 to 2.1), respectively, with no statistically significant difference (p=0.490) (Table 3).

**Table 3:** The CD4/CD8 ratio distribution in women with recurrent miscarriages and IVF-ET failures.

		Total	Miscarriages	IVF-ET Failures
		N=188	N=78	N=110
Mean		1.8	1.8	1.8
Median		1.7	1.7	1.8
SD		0.5	0.5	0.6
Minimum		0.9	1.0	0.9
Maximum		5.5	3.4	5.5
Percentiles	25	1.5	1.5	1.5
	75	2.1	2.1	2.1
Normality test (p-value)	Kolmogorov-Smirnov	0.001	0.001	0.001
	Shapiro-Wilk	0.001	0.001	0.001

In the total population, the mean HLA-DQA1 couple compatibility was 59.7% (± 17.2%), with a median value of 60% (50% to 70.8%). The mean (%) pair HLA compatibility rate was 58.4% (± 15.8%) and 60.5% (± 18.1%) among women with miscarriages and those with IVF-ET failures, respectively, with median values of 57.5% (50% to 65.5%) and 61% (49.3% to 71%), respectively, with no statistically significant difference (p=0.144) (Table 4).

**Table 4:** The distribution of the (%) HLA-DQA1 couple compatibility in recurrent miscarriages and IVF-ET failures.

		Total (%)	Miscarriages (%)	IVF-ET Failures (%)
		N=188	N=78	N=110
Mean		59.7	58.4	60.5
Median		60	57.5	61
SD		17.2	15.8	18.1
Minimum		17	22	17
Maximum		100	100	100
Percentiles	25	50	50	49.3
	75	70.8	65.5	71
Normality test (p-value)	Kolmogorov-Smirnov	0.027	0.003	0.002
	Shapiro-Wilk	0.040	0.042	0.031

The proportion of couples with high HLA-DQA1 allele compatibility (>50%) was 132/188 = 70.2% overall, with no statistically significant difference between couples with miscarriages (51/78 = 65.4%) and those with IVF-ET failures (81/110 = 73.6%) (p=0.222).

Among all infertile women, the frequency of HLA-DQA1*5 allele carriers was 109/188 = 58.0%, with no statistically significant difference between women with miscarriages (41/78 = 52.6%) and those with IVF-ET failures (68/110 = 61.8%) (p=0.206).

Overall, in 79 couples (42.0%), both spouses were HLA-DQA1*5 non-carriers; in 41 cases (21.8%), one of the two spouses was an HLA-DQA1*5 allele carrier, whereas in 68 couples (36.2%) both spouses were HLA-DQA1*5 allele carriers. There was no statistically significant difference in the distribution of the carriage of the HLA-DQA1*5 allele between the couples with miscarriages and those with IVF-ET failures (p=0.734) (Table 5).

**Table 5:** The distribution of the HLA-DQA1*5 allele in the spouses in couples with recurrent miscarriages and IVF-ET failures.

Number of HLA-DQA1*5 allele carriers	Total		Miscarriages		IVF-ET failures	
	N=188		N=78		N=110	
	N	(%)	N	(%)	N	(%)
None	79	42.0	37	47.4	42	38.2
One	41	21.8	17	21.8	24	21.8
Both	68	36.2	24	30.8	44	40

Positive statistically significant correlations were found between the (%) NK cell population and the CD4/CD8 ratio in the whole population (rho = 0.248, p<0.001) and the population of women with IVF-ET failures (rho = 0.297, p=0.002), but not in the group of women with miscarriages (rho = 0.162, p=0.157).

Furthermore, there was a statistically significant positive association of the CD4/CD8 ratio with the couple's HLA-DQA1 compatibility in the cases of miscarriages (rho = 0.266, p=0.019) but not in the cases of IVF-ET failures (rho = -0.094, p=0.331).

Finally, in miscarriages (rho = 0.043, p=0.709) or IVF-ET failures (rho = -0.011, p=0.911), the (%) NK cell population was not associated with HLA pair (%) compatibility. 

No statistically significant difference was found in the (%) percentage of NK cells between carriers and non-carriers of the HLA-DQA1*5 allele in women with miscarriages (p=0.101) or in those with IVF-ET failures (p=0.669). Similarly, there was no statistically significant association between the presence of the HLA-DQA1*5 allele and the CD4/CD8 ratio in the miscarriage group (p=0.128) or the group of IVF-ET failures (p=0.453).

**Figure 1:**
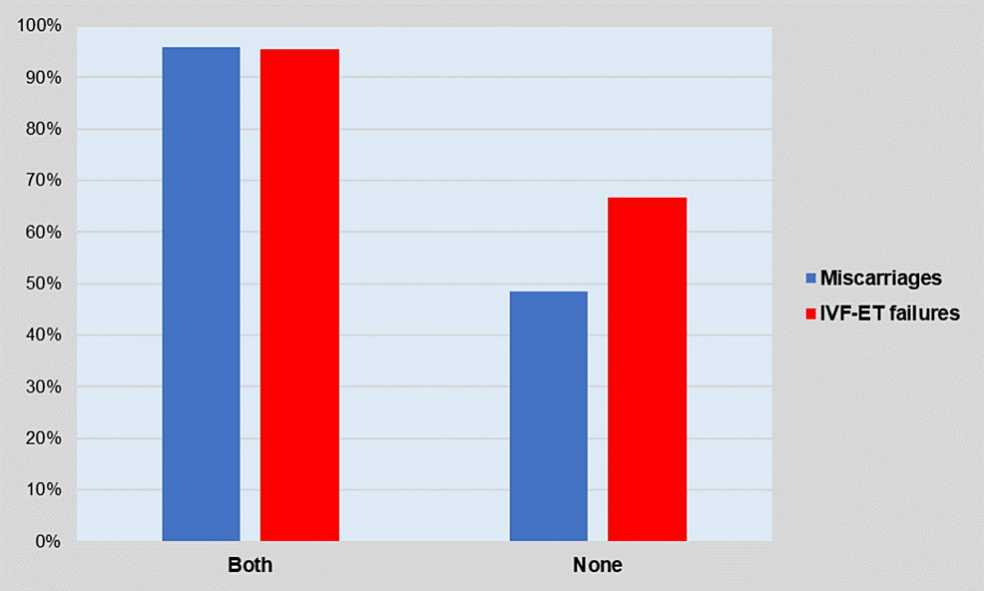
Proportion of couples with high HLA-DQA1 compatibility (>50%) according to the carriage of the HLA-DQA1*5 allele in the spouses.

In contrast, statistically significant associations were found between the presence of the HLA-DQA1*5 allele in subfertile subjects and the probability of high (>50%) HLA-DQA1 couple compatibility. Specifically, compared with the couples in which neither of the spouses carried the allele, those in which both spouses were carriers of the HLA-DQA1*5 allele had a statistically significantly increased probability of high (>50%) HLA-DQA1 compatibility in the total population (65/68 = 95.6% vs. 46/79 = 58.2%, OR = 15.5, 95% CI: 4.5 to 53.7, p<0.001), in the miscarriage group (23/24 = 95.8% vs. 18/37 = 48.6%, OR = 24.3, 95% CI: 3.0 to 198.9, p<0.001), and the IVF-ET failure group (42/44 = 95.5% vs. 28/42 = 66.7%, OR = 10.5, 95% CI: 2.2 to 49.8, p<0.001) (Figure 1).

## Discussion

In this cross-sectional study, key parameters of the immunophenotypic as well as the immunogenetic profiles of a sample of infertile couples with miscarriages and recurrent IVF-ET failures were evaluated.

In our sample, it was found that more than half of women had >10% NK lymphocytes, while 25% had >12.5%, with no statistically significant difference between women with miscarriages and those with IVF-ET failures. NK cells are characterized by their ability to exert cytolytic function against a variety of foreign cells, including neoplastic and virus-infected cells, following the activation of NK cell receptors by HLA class I and II molecules [12]. Furthermore, NK cells are the predominant type of lymphocytes in utero, migrating early in pregnancy from the bone marrow in response to estrogen and progesterone, and they have a central role in trophoblastic infiltration and endometrial modification. An increased proportion of NK cells has been observed in decidua in both animal models and in women with recurrent miscarriages. Peripheral blood NK cells are used as a marker of this lymphocytic activation, although they do not fully correlate with uterine cells [9,13]. The normal proportion of NK cells is around 10% of the total peripheral blood lymphocytes, and NK cell percentages above this value have been associated with poor reproductive outcomes. According to the results of a meta-analysis, increased numbers of peripheral NK cells, as well as higher peripheral NK cell percentages were observed in infertile women and women with recurrent miscarriages, compared with controls; however, in two prospective observational studies, no statistical significant relationship was shown between increased NK cells and live birth rate [10].

The CD4/CD8 ratio was also evaluated and found to be quite high in the whole population, with no difference between the group of women with miscarriages and those with IVF-ET failures. Specifically, in all subjects, the median value of the CD4/CD8 ratio was 1.7, while in almost a third of the women, the CD4/CD8 ratio had a value of 2 or higher. Normally, CD4 T helper cells stimulate the response of other T lymphocytes as well as the humoral immune response. In contrast, suppressive T cells, which carry the marker CD8, are responsible for stopping both humoral and cell-mediated immune responses when necessary. The median CD4/CD8 ratio is normally 1.4-1.5, whereas values above two are considered high. The CD4/CD8 ratio expresses the balance between immune competence and immune stimulation. It is altered in autoimmune disorders and has been hypothesized to be important in the successful adaptation of maternal immunity during pregnancy, indicating that the CD4/CD8 ratio in blood could be a promising immunophenotypic marker for human reproduction [14,15]. The levels of CD4 T cells have been noted to be decreased in the decidua and peripheral blood of women with miscarriages compared with fertile controls [9,16].

Furthermore, in our sample, we found an increased degree of HLA-DQA1 allele similarity between husband and wife. Specifically, in >70% of couples, there was a high degree (>50%) of HLA-DQA1 similarity between spouses, similarly in couples with miscarriages and those with IVF-ET failures. HLA-DQA1 couple coincidence creates similarity between maternal and fetal tissues, and this results in a lack of maternal immune activation necessary for successful implantation and initial fetal development [7,17,18].

In our sample, the CD4/CD8 ratio was statistically significantly positively correlated with the (%) NK cell proportion in the total population and among women with IVF-ET failures and with the (%) HLA-DQA1 sharing in the group with miscarriages.

Additionally, the prevalence of the HLA-DQA1*5 allele was found to be elevated in our sample since the majority (>50%) of the infertile women were carriers. The HLA-DQA1*05 allele is carried by 20%-40% of Europeans and has been strongly associated with autoimmune disorders, including celiac disease and inflammatory bowel diseases, whereas it has also been reported that the carriers tend to develop antibodies against infliximab [19]. In the context of considering the pathogenesis of unexplained miscarriages and implantation failures as a result of systemic or local autoimmunity, the HLA-DQA1*5 allele has been implicated as a risk factor for these disorders [20-22]. We found that the presence of the HLA-DQA1 allele was strongly associated with the total HLA-DQA1 allele compatibility in couples. In our study, the presence of the HLA-DQA1*5 allele in both spouses increased the probability of high (>50%) overall HLA-DQA1 couple compatibility by a factor of 24.3 in the miscarriage group, whereas the corresponding odds were 10.5-fold higher for the IVF-ET failure group. Thus, the carriership of the HLA-DQA1*5 allele could be used as a marker of the degree of HLA-DQA1 compatibility between the couple.

There is accumulating evidence that recurrent miscarriages, as well as IVF-ET failures, share extensive common pathogenetic mechanisms. We have previously reported that genetic heterogeneity of platelet glycoproteins Ia and IIIa associated with platelet-derived thrombophilia is a risk factor for recurrent IVF-ET failures [23], and subsequently, this finding was also confirmed for spontaneous miscarriages [24]. Similarly, the associations of proinflammatory PECAM-1 (Leu125Val) and P-Selectin (Thr715Pro) gene polymorphisms with unexplained spontaneous miscarriages as well as IVF-ET failures have been reported [25,26]. In the present study, we discovered a similar immunophenotypic and immunogenetic profile in women who miscarried and those who failed to implant, with increased levels of NK lymphocytes and CD4/CD8 ratio, a high prevalence of HLA-DQA1*5 allele and elevated HLA compatibility between spouses, as well as positive correlations between these parameters. The limitations of our study include the relatively small sample size and the cross-sectional design, without comparisons with controls. However, the strength of our study is the fact that we included a sample of strictly selected healthy couples with previous recurrent miscarriages and IVF-ET failures without obvious known risk factors. Immunological factors are the most plausible and promising parameters in our efforts to understand the complex phenomena behind unexplained recurrent abortions and implantation failures; however, our view remains incomplete. We must gain more clear evidence of causes and diagnostic tests to make successful steps toward therapy [27].

## Conclusions

High proportion of NK lymphocytes and CD4/CD8 ratio, as well as an elevated prevalence of the HLA-DQA1*5 allele, were identified in this sample of women with recurrent miscarriages and IVF/ICSI-ET failures. In addition, a high percentage of similarity among HLA-DQA1 alleles was found in these couples with negative reproductive outcomes. The CD4/CD8 ratio showed positive correlations with the (%) NK cell proportion among women with IVF-ET failures and with the (%) HLA-DQA1 sharing in the group with miscarriages, which translates into a trend of co-existence of immunological risk factors in this population. The strong positive association of HLA-DQA1*5 allele carriage with the overall compatibility of the HLA-DQA1 alleles highlighted in our study suggests that the HLA-DQA1*5 allele could be used as a surrogate marker for evaluating overall immunological compatibility in infertile couples.
